# Massive facial edema and airway obstruction secondary to acute postoperative sialadenitis or "anesthesia mumps": a case report

**DOI:** 10.1186/1752-1947-3-7073

**Published:** 2009-04-29

**Authors:** Franco Cavaliere, Giorgio Conti, Maria Giuseppina Annetta, Angelo Greco, Alessandro Cina, Rodolfo Proietti

**Affiliations:** 1 Institute of Anaesthesia and Intensive Care, Catholic University of the Sacred Heart, Largo Gemelli 8, 00168 Rome, Italy; 2 Institute of Radiology, Catholic University of the Sacred Heart, Largo Gemelli 8, 00168 Rome, Italy

## Abstract

**Introduction:**

A case of massive facial edema and airway obstruction secondary to an acute sialadenitis is described that occurred a few hours after a neurosurgical procedure performed in the prone position. Literature on this topic is reviewed.

**Case presentation:**

A 73-year-old Caucasian woman underwent a right parieto-occipital craniotomy to remove a meningioma. The procedure was performed in the prone position and lasted for 7 hours. One hour after the end of surgery, left submandibular gland swelling was clearly visible and in a few hours, she developed massive facial edema. Imaging (computed tomography and magnetic resonance) showed inflammatory swelling of the submandibular and parotid glands and of the periglandular tissues, undilated excretory ducts, and complete obliteration of the pharynx lumen (pharyngeal mucosa adhered to the endotracheal tube). Analgesics, corticosteroids, and antibiotics were administered. Edema regressed from the 4th postoperative day and the endotracheal tube could be removed on the 7th postoperative day. The patient was discharged from the surgical intensive care unit on the 14th postoperative day and from hospital on the 28th postoperative day.

**Conclusion:**

This is the first case report in which acute postoperative sialadenitis caused complete upper airway obstruction: only the presence of a tracheal tube avoided the need for an emergency tracheostomy. Since edema evolves insidiously, we recommend caution when removing the endotracheal tube in patients who are acutely developing postoperative sialadenitis.

## Introduction

Acute postoperative sialadenitis, commonly known as 'anesthesia mumps', is occasionally observed after general anesthesia [1]-[4]. Parotid or submandibular swelling develops during surgery or, more often, a few hours later and usually resolves in a few days with no sequelae. The etiology has not been fully explained, but possible causes include trauma, infection, hypersensitivity reactions, and obstruction of the glandular excretory ducts by position, calculi, or thickened secretion. Anesthesia mumps are usually regarded as a mild and transitory complication. Patients may complain of light pain and distress, but airway patency is not threatened, nor are reflexes such as swallowing or coughing impaired. Only in one case report did acute salivary gland swelling during anesthesia induction lead to airway obstruction and tracheostomy [5].

We report a life-threatening postoperative sialadenitis that involved the left submandibular and parotid glands and caused massive facial edema and complete airway obstruction. Due to the severity of the complication, complete imaging was obtained.

## Case presentation

A 73-year-old Caucasian woman underwent a right parieto-occipital craniotomy to remove a meningioma. The surgical procedure lasted for 7 hours and was performed in the prone position with the head slightly turned to the right. Her medical history included arterial hypertension, colonic diverticula, hiatal hernia, cystic renal dysplasia, and no known allergies. Anesthesia was induced with propofol (2 mg/kg) and fentanest (1 μg/kg) and maintained with sevoflurane (1 MAC) and fentanest (boli of 50 to 100 μg intravenously); after muscle relaxation with vecuronium bromide (0.1 mg/kg, followed by boli of 1 mg), her airways were secured with an endotracheal tube (internal diameter 8 mm) and the patient was connected to a mechanical ventilator (Servo 300, Siemens-Elema, Sweden). During anesthesia, 2 g of induction cefazolin was given intravenously. At the end of surgery, the patient was moved to the surgical intensive care unit (S-ICU), where she was sedated with propofol (3 mg/kg/h) and connected to a Servo 300 mechanical ventilator (Siemens-Elema, Sweden) in volume controlled modality.

**Figure 1:**
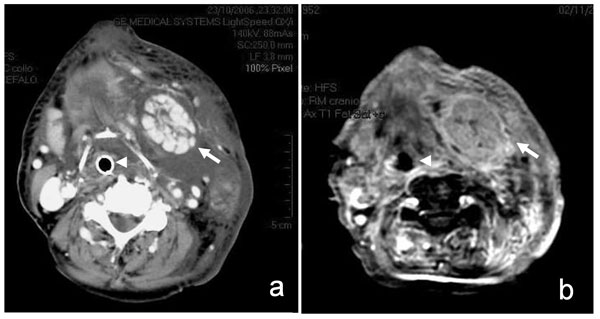
**Enhanced computed tomography (a; 7 hours after the end of surgery) and T1 fat-suppressed magnetic resonance (b; 3rd postoperative day) axial scans of the neck**. The left submandibular gland (arrows) is enlarged, with increased vascularization. The lobular architecture of the gland is conserved and no salivary stones or ductal dilations are evident. A phlegmon is present in the left submandibular space, with obliteration of the fat planes and involvement of the subcutaneous fat. The larynx and trachea are compressed around the tracheal tube (arrowheads) and dislocated to the right side.

One hour later, the patient presented an increasing swelling in the left submandibular triangle which successively spread to the left cheek and to the whole left anterior triangle. Four hours after admission to the S-ICU, no local sign of inflammation was present (the skin was not reddened or warm) and the patient was afebrile. Oral examination revealed massive lingual edema and the presence of petechiae on the gums and on the lateral borders of the tongue. Laboratory tests were normal and serum amylases were 87 IU/L (normal values <100 IU/L). On the basis of the evolving clinical picture, the patient was kept sedated and mechanically ventilated. Drug therapy included diuretics (mannitol 18%, 100 mL intravenously QID), corticosteroids (dexamethasone, 4 mg intravenously TID), and antibiotics (linezolid, 600 mg intravenously BID; meropemen, 1 g intravenously TID; and clindamycin, 600 mg intravenously TID). During the night, serum amylases rose to 230 IU/L and contrast enhanced spiral computed tomography of the head and neck performed 7 hours after the end of surgery showed swelling of the left parotid and submandibular glands with a marked edema that spread to the periglandular fat, muscles, and aponeuroses. Glandular ducts were not dilated and no calculus was observed. Both larynx and pharynx were markedly edematous and the airway lumen was totally obliterated so that no empty space was observed around the endotracheal tube (Figure 1a).

On the 1st postoperative day, the patient was still afebrile. Facial edema had further increased, spreading to the periorbital region and to the forehead. Serum amylases were 211 IU/L. White blood count was normal and semiquantitative procalcitonin measurement was negative (<0.5 μg/mL). Swabs of Wharton's and Stensen's orifices and three blood cultures were performed and the results were negative. Ultrasonography confirmed the swelling of the salivary glands and the absence of calculi, and showed a slight compression of the left internal jugular vein by the enlarged submandibulary gland, but the patency of the vein was maintained; no thrombus was observed in the left subclavian and internal jugular veins.

On the 3rd postoperative day, magnetic resonance imaging of the head and neck was performed with intravenous contrast enhancement (Figure 1b). The exam confirmed the inflammatory process of the parotid and submandibular glands, showing swelling, areas of inhomogeneous signal, and marked enhancement after intravenous contrast. Edema and increased contrast enhancement were also observed in the superficial and deep neck spaces, particularly in the masticatory space. The tongue base was still enlarged and the pharynx and larynx were dislocated to the right.

From the 4th postoperative day, salivary gland swelling and facial edema regressed progressively and on the 7th postoperative day, mechanical ventilation was ended and the endotracheal tube removed. Serum amylases were 111 IU/L on the 9th postoperative day and 116 IU/L on the 13th postoperative day. On the 14th postoperative day, the patient was moved from the S-ICU to the neurosurgical ward. She was fully awake, but still complained of dysphonia and dysphagia; her voice was breathy and she had difficulty in swallowing fluids. In the following days, the patient fully recovered cough and deglutition reflexes. ENT examination indicated the presence of right vocal cord palsy, which was not reported in her medical history; a swelling of the left submandibular gland was still present, but the patient refused surgical biopsy. She was discharged from the hospital on the 28th postoperative day.

## Discussion

Bilateral or unilateral inflammatory swelling of the parotid or submandibular glands can appear during surgery or in the early postoperative period, but does not usually involve the airways, and resolves spontaneously within 5 to 7 days [6]. The peculiarity of the case we report was the massive edema of periglandular tissues which completely obliterated the upper airway lumen.

According to the literature, acute postoperative sialadenitis does not jeopardize airway patency. We could only find one report in which acute pansialadenopathy occurring during anesthesia induction required emergency tracheostomy [5]. In our patient, the onset of life-threatening airway obstruction was insidious because it was progressive and occurred postoperatively, a few hours after the end of surgery. Fortunately, the patient was kept sedated and mechanically ventilated according to internal protocols, otherwise early respiratory weaning and endotracheal tube removal at the end of surgery (when the swelling in the left submandibular triangle was not yet apparent) would have resulted in catastrophic respiratory failure and emergency tracheostomy.

Many potential causes of postoperative sialadenitis have been reported in the literature. Mechanical trauma and parasympathetic nerve stimulation can occur during endotracheal intubation. Excretory duct obstruction by calculi or thickened secretion can cause bacterial infection leading to purulent sialadenitis [6]. In pneumoparotid, increased airway pressure (during ventilation with a facial mask) combined with muscle relaxation causes air to enter the parotid orifice and obstruct the excretory ducts [6]. Adverse drug reactions (usually causing bilateral sialadenitis) can be of type A (augmentation of a pharmacologically known effect) or of type B (abnormal immune-mediated or non-immune-mediated reactions) [7]. A case of acute sialadenitis caused by a type-A drug reaction has been described during morphine infusion [8], while acute sialadenitis by type B reactions has been described during the administration of captopril, nifedipine, and other drugs [7].

The genesis of sialadenitis in our patient was not really explained by the aforementioned causes. Mechanical trauma as a result of endotracheal intubation was unlikely because glandular swelling developed more than 7 hours later. Excretory duct obstruction was ruled out because imaging excluded the presence of air, calculi, or duct dilation. Bacterial sialadenitis was excluded since microbiological cultures were negative and serum procalcitonin was normal. Finally, an adverse drug reaction was also unlikely because fever, skin rash, and eosinophilia were absent; in addition, none of the drugs already associated with drug-induced sialadenitis were administered [7].

In the last few years, several cases of acute sialadenitis have been reported after long-lasting surgical procedures performed in the prone [4] and sitting positions [2], or during head extension [3]. Position may potentially cause glandular ischemia by squeezing arterial or venous vessels, nevertheless, intra-operative venous obstruction can hardly explain our patient because glandular swelling did not occur during surgery. Conversely, arterial ischemia may explain the clinical picture because swelling developed after restoring the supine position (that is to say, after reperfusion). Several cases of acute, transient, ischemic sialadenitis have been described in the literature. One case of ischemic sialadenitis has been reported following facial artery embolization in man [9], and in mice, ischemia of the submandibular gland for several hours induced by arterial ligation and followed by reperfusion is sufficient to cause serious, but reversible glandular damage characterized by necrosis and apoptosis [10].

## Conclusion

We report a peculiar case of postoperative submandibular and parotid sialadenitis in which massive facial and pharyngeal edema resulted in complete obliteration of the upper airway lumen. Hypothetically, intra-operative glandular ischemia may explain the clinical picture. Our report suggests that, in the presence of rapidly developing facial edema in the early postoperative period, a safe artificial airway should be maintained and detailed examination of the upper airway by computed tomography scan should be performed before extubation.

## Abbreviations

ENT: ear nose and throat; MAC: minimum alveolar concentration; QID: quater in die (four times a day); S-ICU: surgical intensive care unit; TID: ter in die (three times a day).

## Consent

Written informed consent was obtained from the patient for publication of this case report and any accompanying images. A copy of the written consent is available for review by the Editor-in-Chief of this journal.

## Competing interests

The authors declare that they have no competing interests.

## Authors' contributions

FC, GC, and RP were responsible for the study conception and design. MGA and AG acquired the data and drafted the manuscript. AC selected and commented on the imaging. All authors have read and approved the final version of the manuscript.
